# Epigenetic DNA Demethylation Causes Inner Ear Stem Cell Differentiation into Hair Cell-Like Cells

**DOI:** 10.3389/fncel.2016.00185

**Published:** 2016-08-03

**Authors:** Yang Zhou, Zhengqing Hu

**Affiliations:** Department of Otolaryngology-Head and Neck Surgery, Wayne State University School of MedicineDetroit, MI, USA

**Keywords:** 5-azacytidine, DNA demethylation, epigenetics, hair cell, methylation, stem cell

## Abstract

The DNA methyltransferase (DNMT) inhibitor 5-azacytidine (5-aza) causes genomic demethylation to regulate gene expression. However, it remains unclear whether 5-aza affects gene expression and cell fate determination of stem cells. In this study, 5-aza was applied to mouse utricle sensory epithelia-derived progenitor cells (MUCs) to investigate whether 5-aza stimulated MUCs to become sensory hair cells. After treatment, MUCs increased expression of hair cell genes and proteins. The DNA methylation level (indicated by percentage of 5-methylcytosine) showed a 28.57% decrease after treatment, which causes significantly repressed DNMT1 protein expression and DNMT activity. Additionally, FM1-43 permeation assays indicated that the permeability of 5-aza-treated MUCs was similar to that of sensory hair cells, which may result from mechanotransduction channels. This study not only demonstrates a possible epigenetic approach to induce tissue specific stem/progenitor cells to become sensory hair cell-like cells, but also provides a cell model to epigenetically modulate stem cell fate determination.

## Introduction

In mammals, inner ear sensory hair cell damage is a major health problem causing deafness or hearing impairments to ~10% of the population, which are usually induced by loud sound, aging, aminoglycoside drug exposure, diseases, and genetic disorders (Basner et al., 2015; Parker and Bitner-Glindzicz, 2015; Wong and Ryan, 2015). Previous studies revealed that non-mammalian vertebrates have the capability to repair injured sensory hair cells; however, this self-repair ability is severely limited in mammals (Jia et al., 2009; Oesterle, 2013). Recent studies suggest that stem cells may be a possible cell source for mammalian sensory hair cell regeneration (Okano and Kelley, 2012; Ronaghi et al., 2012). For instance, mouse embryonic stem cells have been induced to non-neural ectoderm, preplacodal ectoderm, and otic vesicle epithelia, which eventually develop into cell clusters containing hair cells and supporting cells (Koehler and Hashino, 2014). Tissue specific stem/progenitor cells have been isolated from adult mouse utricle sensory epithelia and expressed markers shown in prosensory cells, which are hair cell stem cells during otic vesicle development. These inner ear stem/progenitor cells have shown the capability to differentiate into cells expressing hair cell genes *Myo7a* and *Pou4f3* (Kelley, 2006; Savary et al., 2007; Koehler and Hashino, 2014). Our previous study has demonstrated that adult mouse utricle sensory epithelial cells are able to become prosensory-like cells (MUCs; Zhang and Hu, 2012), which express the genes that are shown in hair cell progenitor cells (Kelley, 2006), suggesting that MUCs may be a valuable cell source to study mammalian hair cell regeneration. For the purpose of future clinical applications to replace human sensory hair cells, it is ideal to guide stem cells to become sensory hair cells without changing DNA sequence. However, it remains unclear how to efficiently achieve this research aim and the mechanism critical for cell differentiation is still obscure.

DNA methylation/demethylation is one of the major epigenetic modifications to regulate gene expression without changing DNA sequence (Jones and Takai, 2001; Jaenisch and Bird, 2003). DNA methylation is a process of adding methyl group to 5-cytosine catalyzed by DNA methyltransferase (DNMT). In mammals, DNMT family has three major members including DNMT1, DNMT3a, and DNMT3b. DNMT1 is responsible for maintenance of methylation pattern through DNA replication, whereas DNMT3a and DNMT3b take charge of the *de novo* DNA methylation. DNA methylation by addition of methyl group to the promoter sequence leads to gene silence, whereas DNA demethylation by removing methyl group from the promoter region of the silenced gene stimulates gene expression (Sanz et al., 2010; Guo et al., 2014). For example, DNA methylation inhibits gene expression in a hematopoietic stem cell line OCI-AML3 by adding methyl groups to the promoter region and transcription start site. However, DNA demethylation of OCI-AML3 activates gene expression by decreasing the genomic methylation level (Lund et al., 2014). It has been reported that DNA demethylation is involved in lineage specification in mouse neural stem cells (Wheldon et al., 2014) and reprogramming of mouse somatic cells into pluripotent stem cells (Chen et al., 2015). These pioneer studies suggest that DNA demethylation plays a critical role in stem cell fate determination. However, previous reports have only studied the relationship between DNA demethylation and gene expression. It is still unclear whether DNA demethylation is able to stimulate the differentiation of stem cells, trigger the expression of differentiation proteins and generate functional differentiated cells.

In our previous study, we treated MUCs with the DNMT inhibitor 5-aza-2′-deoxycytidine (5-aza-CdR) and found that the genomic methylation level was significantly decreased (Zhou and Hu, 2015). 5-aza-CdR treated MUCs increased expression of epithelial genes, hair cell genes, and prosensory genes. However, 5-aza-CdR did not significantly affect the protein expression of epithelial sensory hair cell markers E-cadherin, Cytokeratin, Myosin VI, and Myosin VIIa, which may be a possible explanation for incomplete hair cell differentiation. Moreover, expression of *DNMT1* gene was not significantly changed after 5-aza-CdR treatment. Therefore, additional study and alternative approaches are necessary to guide MUCs to undergo a more complete hair cell differentiation at the protein expression and functional levels.

It has been reported that 5-aza-CdR can only incorporate into DNA and irreversibly binds to DNMT to reduce the addition of methyl groups to DNA (Liyanage et al., 2013; Hackanson and Daskalakis, 2014). There is no evidence showing that 5-aza-CdR has direct effects on protein expression. 5-azacytidine (5-aza) is another DNA methyltransferase inhibitor, which is able to incorporate into both genomic DNA and RNA (Aimiuwu et al., 2012; Borodovsky et al., 2013). The incorporation of 5-aza into DNA shares the similar mechanism of 5-aza-CdR incorporation into DNA. However, 5-aza is primarily incorporated into RNA rather than DNA, by which triggers polyribosome disassembly and defective methylation of transfer RNA, and repress DNMT protein production (Schneider-Stock et al., 2005; Chen et al., 2013). 5-aza has been used to regulate expression of tumor suppressor gene in myelodysplastic syndromes (MDS) and acute myeloid leukemia (AML; Kimura et al., 2012; Orskov et al., 2015), and it has been applied to stem cell research (Rosca and Burlacu, 2011). For instance, 5-aza causes considerable demethylation in the promoter region of osteogenic specific genes, and stimulates mouse bone marrow derived mesenchymal stem cells to differentiate into osteoblasts (Zhou et al., 2009). Additionally, 5-aza regulates expression of bone morphogenetic protein-4 (BMP4) in mouse pluripotent stem cells, which induces adipocyte lineage differentiation (Bowers et al., 2006). These studies suggest that 5-aza may affect the methylation level of stem/progenitor cells to influence stem cell fate determination.

DNA demethylating agents including the DNMT inhibitor 5-aza work best on proliferating cells; therefore, we apply 5-aza to proliferating cell lines such as MUCs in this study. We aimed to investigate whether 5-aza would be able to affect genomic methylation level to stimulate the expression of sensory hair cell genes and proteins. Moreover, we planned to explore the molecular mechanism underlying 5-aza-caused hair cell differentiation using genomic methylation quantification, nested methylation specific PCR (nested-MSP) targeting the promoter region of studied genes, methylated DNA immunoprecipitation (MeDIP), and DNMT activity assays.

## Materials and methods

### Cell culture and DNA demethylation treatment

Mouse utricle sensory epithelia-derived progenitor cell lines (MUC), which have been established in our previous study (Zhang and Hu, 2012), were used in this study. MUCs were cultured in DMEM/F12 GlutaMAX™ with 10% fetal bovine serum (FBS, all from Invitrogen) in an incubator supplied with 5% CO_2_ at 37°C (Zhang and Hu, 2012). 5-azacytidine (5-aza, Sigma) was applied to MUC when the cell confluence reached 40–50%. MUCs in the control group were cultured in the culture medium in the absence of 5-aza. Control and treated MUCs were observed daily using phase contrast microscopy and images were captured using a digital camera. MUCs were treated with 5-aza for 5 days, and MUCs were passaged when cell confluence reached 70–80%.

### Viability assay

To test the cytotoxicity of 5-aza, MUCs were exposed to 20, 40, 80, 160, and 320 μM of 5-aza for 48 h. In the control group, MUCs were kept in the medium containing vehicle (DMEM/F12). After 48 h of 5-aza treatment, 2 μM calcein AM, and 0.3 μg/ml propidium iodide (PI, both from Invitrogen) were added to culture medium for 30 min, followed by Hoechst 33,342 (10 μg/ml, Invitrogen) staining to label nuclei. MUCs were observed by epifluorescence microscopy with a cooled CCD camera (*n* = 5/group). One-way analysis of variance (ANOVA) and Tukey's *post-hoc* test were used for analysis and *P* < 0.05 was determined as the criterion of statistical significance in this study.

### RNA extraction, reverse transcription PCR (RT-PCR), and real time quantitative RT-PCR (quantitative PCR)

After 5 days of 5-aza treatment, total RNA was extracted from the control and treated MUCs with an RNeasy Mini Kit (Qiagen), followed by cDNA conversion using a QuantiTect Reverse Transcription Kit (Qiagen) according to manufacturers' protocols. A thermal cycler (Eppendorf) was used for RT-PCR with GoTaq® Green Master Mix (Promega) and primers in Table 1. PCR products were electrophoresed and imaged using a ChemiDoc-It® 2 imaging system (UVP).

**Table 1:** **Primers used in RT-PCR, quantitative PCR, Nested-MSP and MeDIP-quantitative PCR**.

**Gene**	**Forward: 5′-3′**	**Reverse: 5′-3′**	**Product length (bp)**
*Gapdh*	GGCCGCATCTTCTTGTGCAGT	TGCAAATGGCAGCCCTGGTGA	111
*Cdh1*	ATTCAAAGTGGCGACAGACGGC	ACCTGGGTACACGCTGGGAAACAT	223
*Krt8*	CAAGGTGGAACTAGAGTCCCG	CTCGTACTGGGCACGAACTTC	187
*Snai1*	ATGCACATCCGAAGCCACACG	TGGAGCAAGGACATGCGGGAGAA	245
*Snai2*	CATCCTTGGGGCGTGTAAGTC	GCCCAGAGAACGTAGAATAGGTC	186
*Zeb1*	ACTGCAAGAAACGGTTTTCCC	GGCGAGGAACACTGAGATGT	127
*Zeb2*	CCACGCAGTGAGCATCGAA	CAGGTGGCAGGTCATTTTCTT	131
*Vim*	AAGCCGAAAGCACCCTGCAGTCAT	AGGTCAGGCTTGGAAACGTCCACA	202
*Fn1*	GTGACACTTATGAGCGCCCTA	CCACTTGTCGCCAATCTTGTA	137
*Cdh2*	ATGCCCTGAATGGAATGCTGCGGT	GCTGTGGCTGTGTTTGAAAGGCCA	211
*Bmp4*	CATGAGGGATCTTTACCGGCTC	TCTCCAGATGTTCTTCGTGATGG	140
*P27*kip*1*	GCGGTGCCTTTAATTGGGTC	TTCGGGGAACCGTCTGAAAC	197
*Prox1*	TGGAGAAGTATGCGCGTCAA	CTGCGCAACTTCCAGGAATC	155
*Lfng*	GCCGTCAAGACCACCAGAAAG	GGTCATACTCCACAGCCATCTT	208
*Sox2*	GCGGAGTGGAAACTTTTGTCC	CGGGAAGCGTGTACTTATCCTT	157
*Dlx5*	CACCACCCGTCTCAGGAATC	GCTTTGCCATAAGAAGCAGAGG	125
*Notch1*	GCGTCACTTGGCAGCCTCAA	CACCCCACAGCCCACAAAGA	144
*Hes1*	AGCACAGAAAGTCATCAAAGCC	ATGTCTGCCTTCTCTAGCTTGG	142
*Hes5*	AGTCCCAAGGAGAAAAACCGA	GCTGTGTTTCAGGTAGCTGAC	183
*Isl1*	ATGATGGTGGTTTACAGGCTAAC	TCGATGCTACTTCACTGCCAG	174
*Jag1*	CCTCGGGTCAGTTTGAGCTG	CCTTGAGGCACACTTTGAAGTA	150
*Numb*	CTTCCCAGTTAAGATCCTCGGC	CCCGTTGTTCCAAAGAAGCCT	126
*Atoh1*	CCCGTCCTTCAACAACGACAAG	AGGTGATGGTGGTCATTTTTGC	156
*Myo7a*	GCACTTCATCATCGGCAACGGCAT	GCTGCTCTTGGATGGGTTGTGTGT	100
*Myo6*	GGCATCGTCCCAAGAGATTTTC	CCACAATGTCAAAGTTCGGTACA	150
*Espn*	CTGCCTGGAGACGAGACATT	GCAGCTTCTCCGACTGTTCT	123
*Pou4f3*	CGCACAACAACATGATCGCT	TTCTCGGATGAAGGACGTGG	199
Methylated *Cdh1*	GTTTTTAGTTAATTAGCGGCGTC	ACACTAAACTCGAATACGATCGAA	175
Unmethylated *Cdh1*	GTTTTTAGTTAATTAGTGGTGTTGG	CACTAAACTCAAATACAATCAAA	174
Methylated *Atoh1*	TATTTTGTAGGCGAGAGATTTTTTC	ACTCACCCTAACTATCAACCTCGT	197
Unmethylated *Atoh1*	TTTTGTAGGTGAGAGATTTTTTTGT	AACTCACCCTAACTATCAACCTCAT	196
Methylated *Pou4f3*	GCGGTTTCGTTTTGTTTTTC	GTTCATAACCATCATCTTCGAA	263
Unmethylated *Pou4f3*	GATTTGTGGTTTTGTTTTGTTTTTT	AACATTCATAACCATCATCTTCAAA	271
MeDIP *Cdh1*	TTTAGTTAGTAAAGGTTAATGGCGG	AAACTTTATTCATCATCTAAATTTCCGT	268
MeDIP *Atoh1*	TTTTTTTGATTGGGTAGATACGC	CTCCGATTACTAAAAACGCTACG	117
MeDIP *Pou4f3*	GCGGTTTCGTTTTGTTTTTC	GTTCATAACCATCATCTTCGAA	263

Quantitative PCR was performed by Bio-Rad CFX system with SsoAdvanced™ SYBR® Green Supermix (Bio-rad) (*n* = 3). The melting temperature and efficiency of primers have been determined in a previous study (Zhou and Hu, 2015). The mean quantification cycle (Cq) was calculated by Bio-Rad CFX Manager software in a regression mode and studied genes with Cq value >40 was believed no expression and excluded from statistical analysis. The Cq values of the internal control gene *Gapdh* in the control and treated groups that had been calibrated within one cycle were used as calibrator references for analysis. The 2^−ΔΔCq^ (Livak) Method (Bio-Rad CFX Manager software) was used to quantify the fold change of studied genes. In quantitative analysis, relative mRNA expression change ≥2-fold was considered to be biologically important and qualified for statistical analysis, and a two-tailed Student's *t*-test was used for statistical analysis.

### Genomic DNA extraction, bisulfite conversion reactions, and nested-methylation-specific PCR (nested-MSP)

Genomic DNA (gDNA) was extracted from MUCs from the control and treatment groups using a Flexi DNA Kit (Qiagen), followed by a bisulfite conversion reaction using an EpiTect Bisulfite Conversion Kit (Qiagen) according to the manufacturer's protocol. Nested-MSP was used to detect the methylation pattern of studied genes with a Qiagen MSP Kit (*n* = 3). The primers targeting the CpG islands of promoter regions of studied genes for nested-MSP were included in Table 1. The products of nested-MSP were analyzed by electrophoresis and ChemiDoc-It® 2 imaging system. To compare the methylation level of studied genes, expression of unmethylated studied gene was normalized in the control and treated MUCs.

### Methylated DNA immunoprecipitation (MeDIP) and quantitative PCR

Before DNA shearing, gDNA of both control and treated MUCs were diluted to a final concentration of 100 μg/ml. Digital sonifier 250 (Branson) was used to shear the gDNA by 5 pulses of sonication at 205 amplitude for 10 s, followed by immunoprecipitation using an EpiQuik™ Methylated DNA Immunoprecipitation Kit (Epigenetek) according to the manufacturer's protocol. The same amount of gDNA fragments from the control and treatment groups was added to the immunoprecipitation reaction. Quantitative PCR was used to quantitatively evaluate the amount of methylated gene using the same amount of MeDIP product templates of the control and treatment MUCs (*n* = 3). The primers targeting the CpG islands of promotor regions of studied genes for MeDIP quantitative PCR were shown in Table 1. Quantitative amount of methylated gene was described by the mean quantification cycle (Cq), which was calculated by Bio-Rad CFX Manager software. In this study, the mean Cq value change ≥1 cycle (which means at least two-fold expression changes) was considered to be of biomedical importance. The fold change of studied genes (ΔΔCq) was calculated using the 2^−ΔΔCq^ (Livak) Method (Bio-Rad CFX Manager software). A two-tailed Student's *t*-test was used to compare the mean Cq values of studied genes between the control and treatment groups. Cq values >40 were not considered to have significant gene expression thus excluded from statistical analysis.

### Immunofluorescence

After 5 days of 5-aza treatment, control and treated MUCs were fixed in 4% paraformaldehyde for 10 min at room temperature, followed by incubation in PBS containing 5% donkey serum (Jackson Immunoresearch), and 0.2% Triton X-100 (Sigma) for 20 min. Primary antibodies included epithelial markers anti-E-cadherin (1:200; Santa Cruz) and anti-pan-cytokeratin 26 (PCK26; 1:100; Sigma), and hair cell markers anti-Myosin VI (Myo6; 1:200; Sigma), anti-Myosin VIIa (Myo7a; 1:200; DSHB), anti-Math1 (Atoh1; 1:200; DSHB), anti-Pou4f3 (1:200; Sigma), anti-Espin (1:100; a gift from Dr. James Richard Bartles, Northwestern University), anti-DNMT1 (1:100; Epigentek) and anti-PCNA (1:100; Abcam). Secondary antibodies were Dylight-488-, Dylight-549,- and Dylight-649-conjugated antibodies, with a universal nuclear marker, 4′,6-diamidino-2-phenylindole (DAPI, all from Jackson Immunoresearch). MUC samples were incubated with primary antibodies overnight at 4°C, followed by secondary antibodies incubation for 2–3 h at room temperature. MUCs were observed using a Leica epifluorescence microscope and images were captured using a QImaging monochrome cool CCD camera.

### Nuclear extraction and western blotting

The nuclear extracts from the control and treated MUCs were harvested using a Chemicon Nuclear Extraction Kit (Millipore) according to the manufacturer's protocol. Western blotting was used to study the protein expression in the control and treated MUCs (*n* = 3). Antibodies used in western blotting included monoclonal goat anti-E-cadherin (E-cad, 1:100; Santa Cruz), polyclonal rabbit anti-Myosin VI (1:100; Sigma), monoclonal mouse anti-Myosin VIIa (1:100; DSHB), polyclonal rabbit anti-Pou4f3 (1:200; Sigma), monoclonal mouse anti-DNMT1 (1:100; Epigentek), and mouse anti-GAPDH (1:200; Thermo Scientific). Secondary antibodies included donkey anti-goat HRP-conjugated, donkey anti-rabbit HRP-conjugated, donkey anti-mouse HRP-conjugated antibodies, and HRP standard protein (all from Bio-Rad). Clarity™ ECL Western Blotting Substrate (Bio-Rad) was applied to blotting membranes, and images were captured by a ChemiDoc-It® 2 imaging system. To quantify protein expression changes, ImageJ (NIH) was used to measure the band density of studied proteins. The protein band density of control MUCs was normalized to 1, and the protein density of treated MUCs was determined by its relative intensity to that of control MUCs.

### Genomic methylated DNA quantification

Genomic methylated DNA was examined using a MethylFlash Methylated DNA Quantification Kit (Epigentek). The positive control of methylated DNA was artificially synthesized and provided with the kit by the manufacturer, in which 50% DNA has been methylated at 5-cytosine. A standard curve was generated by diluting the positive control to a series of concentrations at 0, 0.5, 1, 2, 5, and 10 μg/ml following the manufacturer's protocol. The relative fluorescence unite (RFU) was read at 530_EX_/590_EM_ nm using a Gemini EM fluorescence microplate reader (Molecular Devices). The genomic methylation level was quantified by the percentage of 5-methylcytosin (5-mC%), which was determined as $\frac{\begin{array}{c} \left(\text{sample RFU-negative control RFU}\right)÷\text{amount of input}\\ \text{sample DNA }(\text{ng}) \end{array}}{\begin{array}{c} \left(\text{positive control RFU-negative control RFU}\right)\times 2\;÷\text{amount}\\ \text{of input positive control }(\text{ng}) \end{array}}$ × 100% (*n* = 4). The genomic methylation inhibition was calculated as: $\frac{5-\text{mC}\%\left(\text{experiment}\right)-\;5-\text{mC}\%(\text{control})}{5\text{-mC}\%(\text{experiment})}\;$ × 100%. A two-tailed Student's *t*-test was used to compare the 5-mC% of the control and treatment groups.

### DNMT activity assay

DNMT activity was estimated in the nuclear extracts of the control and treatment group using an EpiQuik DNMT Activity/Inhibition Assay Ultra Kit—Fluorometric (Epigentek) according to the manufacturer's protocol. Gemini EM fluorescence microplate reader was used to read the relative fluorescence unit (RFU) at 530_EX_/590_EM_ nm (*n* = 4). DNMT activity (RFU/h/μg) was determined as: $\frac{(\text{Sample}-\text{Blank RFU)}}{\text{Protein amount }\left(\text{μg}\right)\;\times \text{Reaction time }(\text{h})}$. The DNMT inhibition was calculated as: $\frac{\text{Treated sample RFU}-\text{Blank RFU}}{\text{Control sample RFU}-\text{Blank RFU}}$ × 100%.

### Mechanotransduction channel permeation assay

Functional sensory hair cells usually possess potent mechanosensory transduction channels to convert mechanical movements into cellular activities. A styryl pyridinium dye N-(3-triethylammoniumpropyl)-4-[4-(dibutylamino)styryl] pyridiniumdibromide, FM1-43 has been used to evaluate functional mechanosensory transduction channels in development and stem cell regeneration (Geleoc and Holt, 2003; Hu and Corwin, 2007). In this study, FM1-43 (Invitrogen) was used as a probe to investigate the existence of mechanotransduction channels. In an initial optical assay for mechanotransduction channel function, the control and treated MUCs were incubated in DMEM/F12 containing 5 μM FM1-43 for 5–30 s at room temperature and rinsed by DMEM/F12 for 3 times (*n* = 5). Samples were immediately examined under a Leica epifluorescence microscope and imaged using a QImaging monochrome cool CCD camera. In quantitative study, MUCs showing FM1-43 label around nuclei were quantified for cell counting (*n* = 5).

## Results

### Viability of MUCs following 5-aza treatment

**Figure 1:**
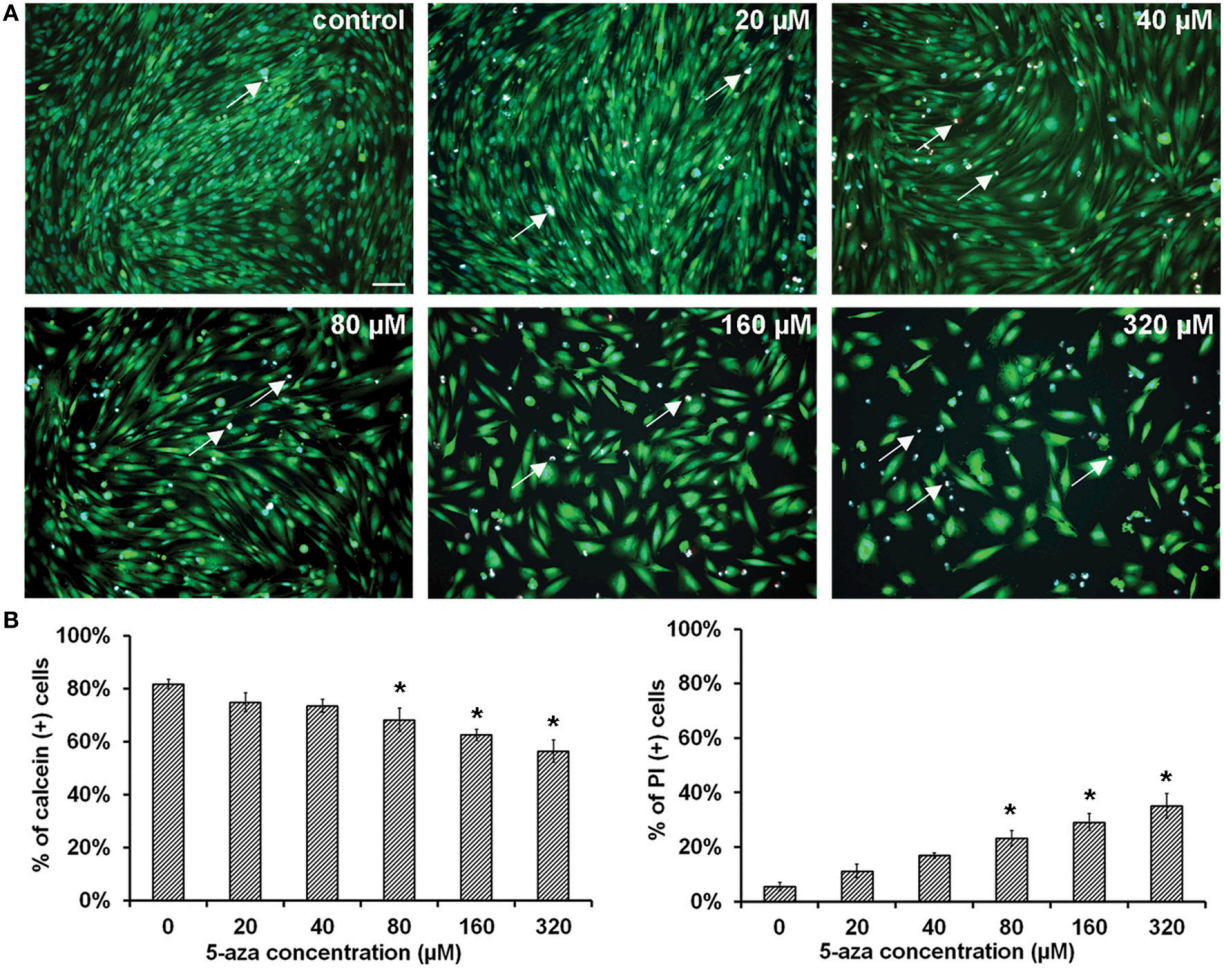
**Viability of MUCs following 5-aza treatment. (A)** Calcein and PI were used to determine the viability of MUCs treated with 0, 20, 40, 80, 160, and 320 μM 5-aza (*n* = 5). Hoechst 33,342 was used to label all nuclei (blue), calcein for viable cells (green), PI for dead cells (red, double labeled with Hoechst 33,342 thus showing in pink, arrows). Scale bar, 100 μm. **(B)** The percentage of viable cells decreased with the 5-aza concentration, whereas percentage of dead cells increased, which was significantly different (*P* < 0.05 ANOVA). Tukey *post-hoc* test suggested that 20–40 μM 5-aza did not cause obvious cell death (*P* > 0.05).

Calcein AM, propidium iodide (PI), and Hoechst 33342 were used to determine the viability of MUCs. After treatment of 5-azacytidine (5-aza) for 48 h, viable MUCs were stained with calcein AM showing green (Figure 1A; *n* = 5). Approximately 81.75 ± 1.72%, 74.86 ± 3.48%, 73.52 ± 2.50%, 68.21 ± 4.41%, 62.48 ± 2.19%, and 56.38 ± 4.26% MUCs were labeled by calcein AM when exposed to 0 (control), 20, 40, 80, 160, and 320 μM 5-aza respectively (Figure 1B; *n* = 5). One-way analysis of variance (ANOVA) showed significant difference among these groups (*P* < 0.05). Comparing with control MUCs, Tukey *post-hoc* test indicated that 80, 160, and 320 μM 5-aza treatments caused a significant reduction in the number of viable MUCs (*P* < 0.05), whereas 20 and 40 μM 5-aza did not have significant effects (*P* > 0.05). The dead MUCs were labeled by PI showing red (Figure 1A). Quantitative analysis showed that 5.56 ± 1.52%, 11.23 ± 2.53%, 16.89 ± 1.01%, 23.27 ± 2.89%, 29.18 ± 3.07%, and 35.08 ± 4.57% MUCs were stained by PI in 0 (control), 20, 40, 80, 160, and 320 μM 5-aza, respectively (Figure 1B), which was significantly different (*P* < 0.05, ANOVA). Tukey *post-hoc* test suggested a significantly increased number of dead MUCs in 80, 160, and 320 μM 5-aza groups (*P* < 0.05). Neither 20 nor 40 μM 5-aza caused obvious cell death (*P* > 0.05). Therefore, 40 μM 5-aza was selected for further studies.

### Expression of prosensory genes following 5-aza treatment

**Figure 2:**
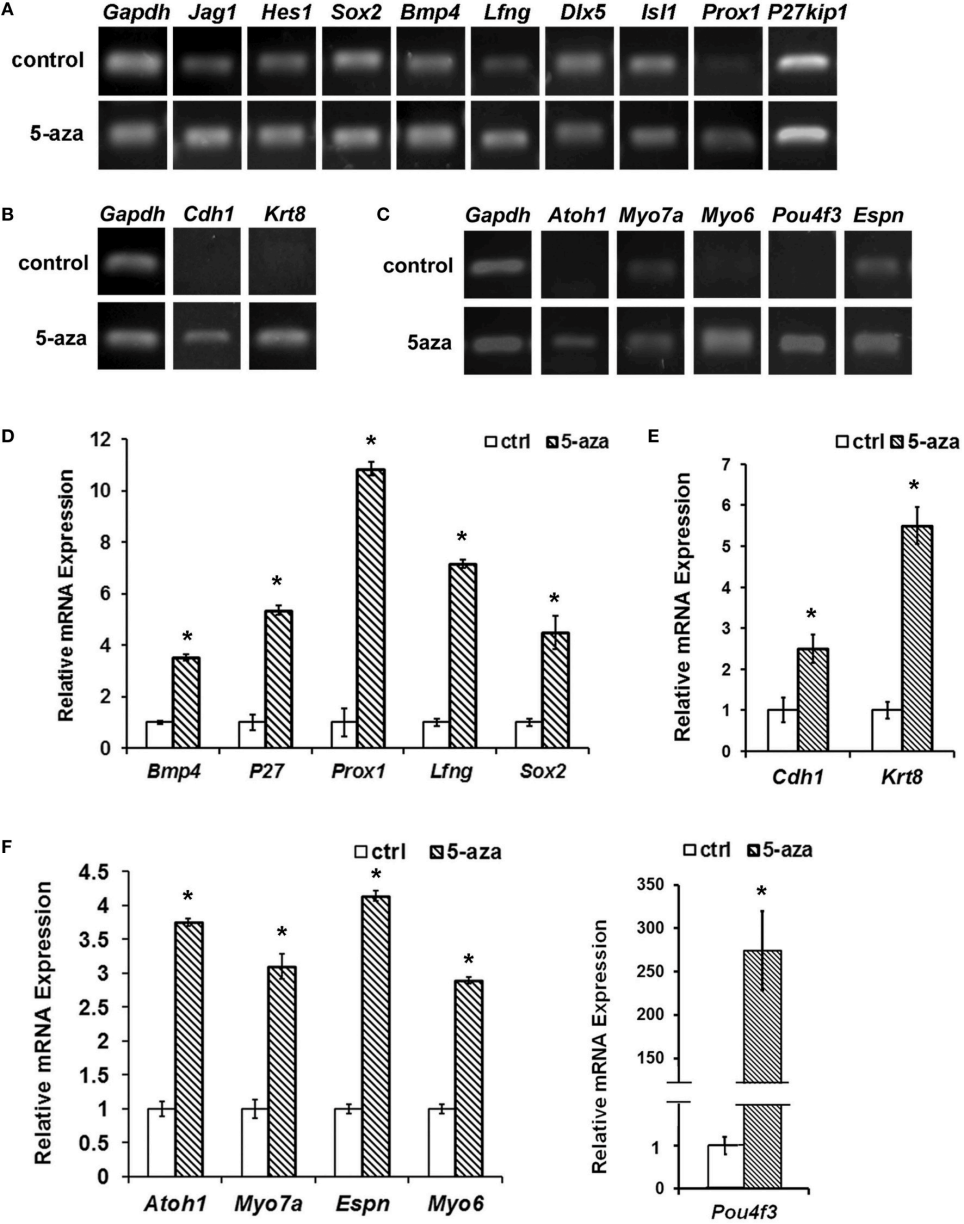
**Gene expression changes following 5-aza treatment. (A)** Following 5-aza treatment, RT-PCR showed increased expression in *Sox2, Bmp4, Lfng, Prox1*, and *P27*^*kip*1^, whereas no obvious change in *Jag1, Hes1, Isl1*, and *Dlx5*. **(B)** RT-PCR showed that MUCs up-regulated expression of epithelial genes *Cdh1* and *Krt18* following 5-aza treatment. **(C)** RT-PCR showed that expression of hair cell genes *Atoh1, Myo7a, Myo6, Espn*, and *Pou4f3* was up-regulated following 5-aza treatment. **(D)** Quantification PCR showed ~3.53 ± 0.11, 5.36 ± 0.17, 10.86 ± 0.27, 7.17 ± 0.17, and 4.49 ± 0.64 fold of up-regulation of *Bmp4, P27*^*kip*1^, *Prox1, Lfng*, and *Sox2*, respectively after 5-aza treatment (*P* < 0.05, Student *t*-test; *n* = 5). **(E)** Quantification PCR showed normalized relative expression of *Cdh1* and *Krt18* of treated MUCs were 2.50 ± 0.35 and 5.50 ± 0.45 fold higher than those of control MUCs, which is statistically significant (*P* < 0.05; Student's *t*-test; *n* = 5). **(F)** Quantitative study indicated that normalized relative expression values of *Atoh1, Myo7a, Myo6, Espn*, and *Pou4f3* were ~5.74 ± 1.27, 3.10 ± 0.19, 1.89 ± 0.07, 4.14 ± 0.07, 274.42 ± 45.49 fold higher than those of control MUCs respectively, and statistical analysis showed significant difference (*P* < 0.05; Student's *t*-test; *n* = 5). ^*^ Indicates significant difference comparing with the control group.

Reverse transcription PCR (RT-PCR) showed that expression of some prosensory genes including *Sox2, Bmp4, Lfng, Prox1*, and *P27*^*kip*1^ was significantly higher in the treatment group than those in the control group (Figure 2A). Quantitative PCR showed 3.53 ± 0.11, 4.49 ± 0.64, 10.86 ± 0.27, 7.17 ± 0.17, and 5.36 ± 0.17 fold of up-regulation of *Bmp4, Sox2, Prox1, Lfng* and *P27*^*kip*1^ respectively, which was statistically significant (Figure 2D; *P* < 0.05, Student's *t*-test). Other studied prosensory genes did not show significant expression changes (≤2-fold), including *Jag1* (1.22 ± 0.04 fold), *Hes1* (1.18 ± 0.04 fold), *Dlx5* (0.55 ± 0.02 fold), and *Isl1* (1.03 ± 0.03 fold).

### Expression of sensory hair cell markers and differentiation of functional hair cell-like cells

In response to 40 μM 5-aza treatment for 5 days, expression of epithelial genes *Cdh1* and *Krt8* was increased comparing with control MUCs (Figure 2B). Quantitative PCR showed that *Cdh1* and *Krt8* had 2.50 ± 0.35 and 5.50 ± 0.45 fold of up-regulation after treatment respectively (Figure 2E). To study whether treated MUCs differentiated into hair cells, expression of hair cell genes was investigated using RT-PCR and quantitative PCR. After treatment, RT-PCR suggested increased expression of hair cell genes including *Atoh1, Myo7a, Myo6, Espn*, and *Pou4f3* (Figure 2C). In quantitative PCR, normalized relative expression of *Atoh1, Myo7a, Myo6, Espn*, and *Pou4f3* was ~3.74 ± 1.27, 3.10 ± 0.19, 2.89 ± 0.07, 4.14 ± 0.07, 274.42 ± 45.49 fold higher than those of control MUCs respectively (Figure 2F). Student's *t*-test indicated significant difference between the control and treatment groups (*P* < 0.05).

**Figure 3:**
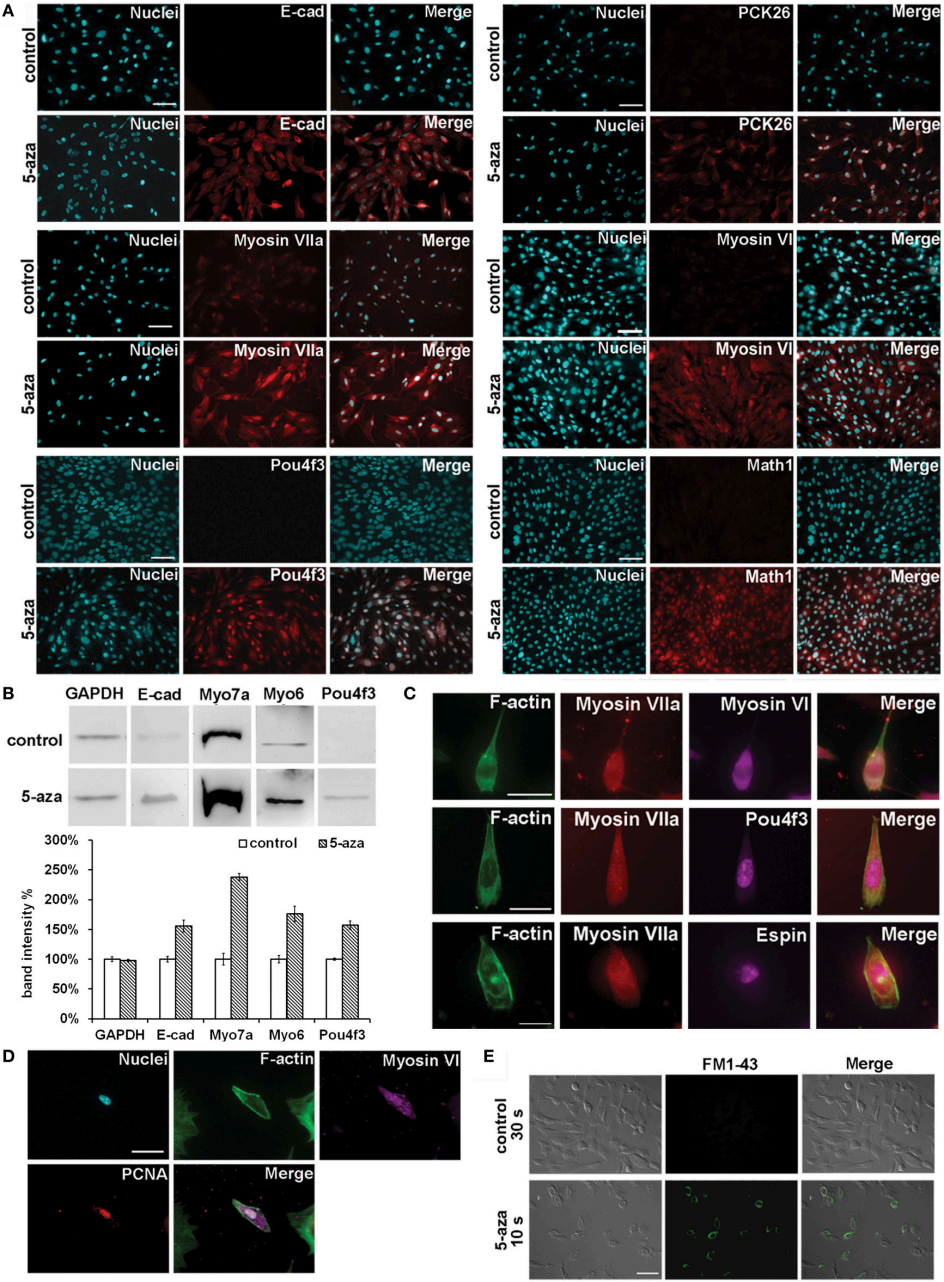
**Hair cell protein expression changes following 5-aza treatment. (A)** Immunofluorescence showed up-regulated expression of epithelial hair cell markers E-cad, PCK26, Myosin VIIa, Myosin VI, Math1, and Pou4f3 in treated MUCs (Scale bar 100 μm). **(B)** Western blotting showed significant increased expression of Myosin VIIa and Myosin VI, and slightly up-regulation of E-cad and Pou4f3 in treated MUCs. **(C)** Multiple-labeling immunofluorescence showed that newly-generated hair cell-like cells were double-labeled with Myosin VIIa and Myosin VI, Myosin VIIa and Pou4f3, and Myosin VIIa and Espin (scale bar 50 μm). **(D)** Hair cell-like cells were PCNA and Myosin VI positive, suggesting that these cells were newly-generated cells (scale bar 50 μm). **(E)** In initial optical assay using FM1-43 permeation test, control MUCs did not show FM1-43 signal after 30 s incubation. In treated MUCs, ~30% of treated MUCs were filled with FM1-43 when they were exposed to FM1-43 for 10 s at room temperature (scale bar 100 μm).

Immunofluorescence indicated that control MUCs did not exhibit expression of E-cadherin (E-cad, encoded by *Cdh1*), pan-cytokeratin 26 (PCK26, encoded by *Krts* including *Krt8*), Math1 (encoded by *Atoh1*), Myosin VI, or Pou4f3, but had weak Myosin VIIa expression. After treatment, immunofluorescence showed that treated MUCs significantly increased protein expression of these sensory hair cell markers (Figure 3A). Additionally, western blotting revealed remarkably increased expression of E-cadherin, Myosin VIIa, Myosin VI, and Pou4f3 in treated MUCs (Figure 3B). To quantify protein expression changes, we measured protein band density using ImageJ, with protein band density of control MUCs normalized to 1. We found that the relative protein densities in treated MUCs were GAPDH 97.71 ± 2.33%, E-cadherin 155.71 ± 9.86%, Myosin VIIa 238.41 ± 6.42%, Myosin VI 176.33 ± 12.96%, and Pou4f3 157.51 ± 7.06% (Figure 3B). These protein studies indicated that 5-aza was able to stimulate MUCs to increase protein expression of hair cell markers. To further ascertain sensory hair cell generation, treated MUCs were double labeled by epithelial hair cell markers, including Myosin VIIa + Myosin VI and Myosin VIIa + Pou4f3 (Figure 3C). Quantitative study showed that 29.04 ± 4.65% of treated MUCs were Myosin VIIa and Myosin VI positive (*n* = 5). Additionally, the hair bundle-like structures were labeled by anti-Espin antibody (encoded by *Espn*; Figure 3C). To examine whether hair cell-like cells are newly-generated cells, anti-PCNA antibodies were used to co-label with anti-Myosin VI antibodies and the result indicated that some treated MUCs were both PCNA and Myosin VI positive (Figure 3D), suggesting that hair cell-like cells were newly-generated cells. These results demonstrated that treated MUCs expressed hair cell proteins and the hair bundle specific protein Espin, indicating that 5aza was able to stimulate MUCs to adopt a hair cell-like cell fate.

Because we found expression of hair cell genes and proteins in treated MUCs, we performed a mechanosensory transduction permeation assay to investigate whether treated MUCs possessed mechanosensory transduction channels, which are specifically shown in sensory hair cells. FM1-43 has been reported to be able to fill into the cytoplasm of hair cells with functional mechanosensory transduction channels when hair cells are briefly exposed to this compound (Geleoc and Holt, 2003). Additionally, FM1-43 permeation assay has been used to determine mechanosensory transduction channels in stem cell studies (Hu and Corwin, 2007). In an initial optical assay, we did not observe FM1-43 positive cells in control MUCs after 30 s of incubation (Figure 3E). In treated MUCs, ~30% of treated MUCs were filled with FM1-43 when they were exposed to FM1-43 for 10 s at room temperature (Figure 3E; *n* = 5). This study revealed that treated MUCs were permeable to FM1-43, suggesting that treated MUCs may possess potential FM1-43-permeation related mechanotransduction channels that are shown in functional sensory hair cells.

### Genomic demethylation, DNMT expression, and enzyme activity assay

**Figure 4:**
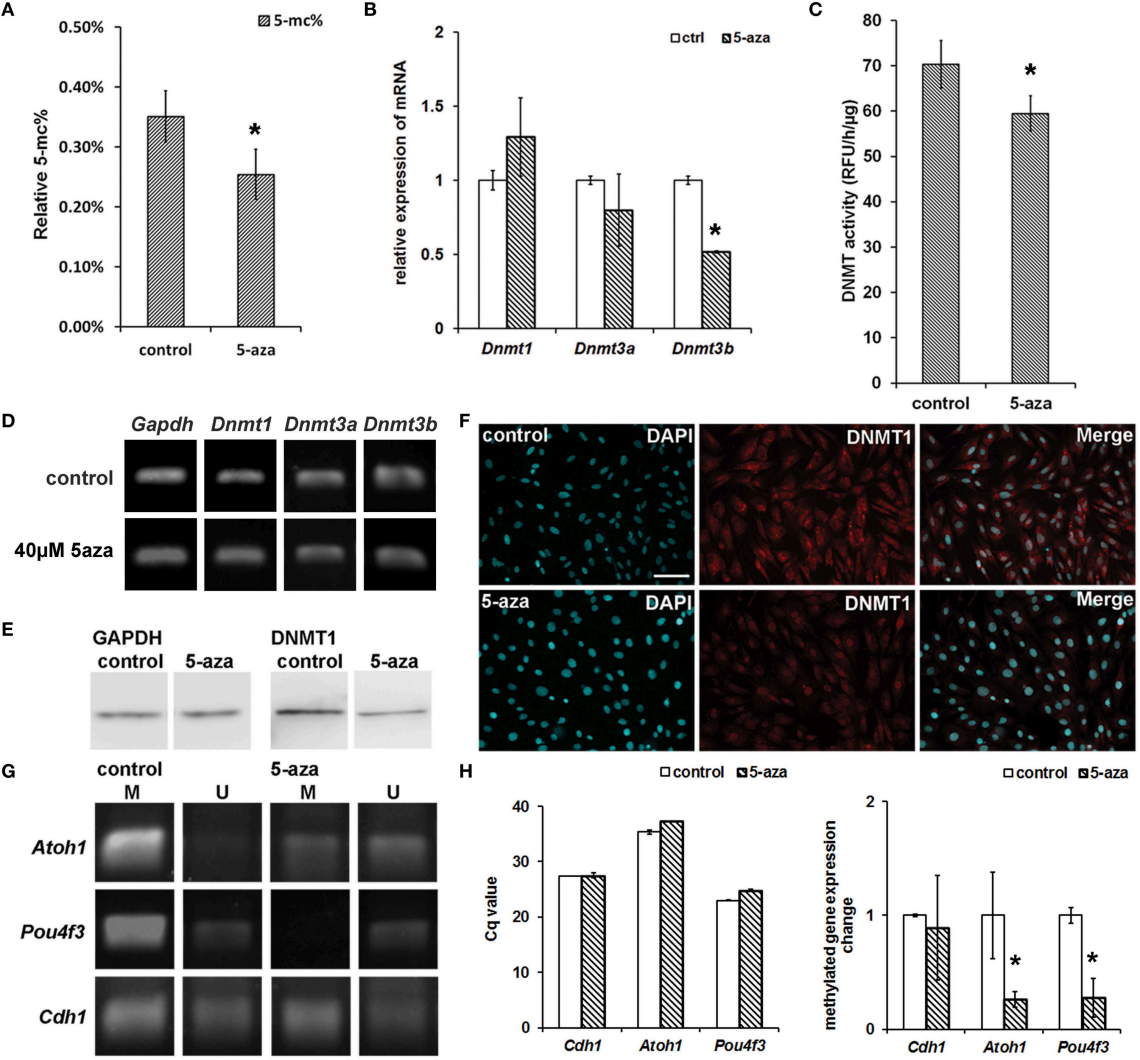
**Genomic demethylation, DNMT1 protein down-regulation and DNMT activity suppression. (A)** 5-aza treatment decreased genomic 5-mC% from 0.35 ± 0.04% (control MUCs) to 0.25 ± 0.04% (treated MUCs), which was ~28.57% decrease (*P* < 0.05, Student's *t*-test; *n* = 5). **(B)** Quantitative PCR showed approximately 1.29 ± 0.26 fold up-regulated *Dnmt1*expression, 0.80 ± 0.24 fold down-regulated *Dnmt3a* expression, and 0.52 ± 0.01 fold down-regulated *Dnmt3b* expression. **(C)** DNMT activity assay revealed the activity of DNMT decreased from 70.33 ± 5.24 RFU/h/μg (control MUCs) to 59.47 ± 3.87 RFU/h/μg (treated MUCs) after 5-aza treatment, which caused 15.37 ± 0.09% inhibition of DNMT activity (*P* < 0.05, Student *t*-test; *n* = 5). **(D)** RT-PCR suggested no obviously change of *Dnmt1* and *Dnmt3a*, but down-regulation of *Dnmt3b* in treated MUCs. **(E)** Western blotting showed significant decreased expression of DNMT1 in treated MUCs. **(F)** Immunofluorescence showed down-regulated expression of DNMT1 in treated MUCs. The DNMT1 expression was restricted to the nuclei after treatment (scale bar 100 μm). **(G)** Nested-MSP targeting the CpG island in promoter region showed that the methylation/unmethylation ratio of studied sensory hair cell markers was significantly decreased after 5-aza treatment (M, methylated DNA; U, unmethylated DNA). **(H)** Compared to control MUCs (normalized to 1), MeDIP-quantitative PCR showed 0.89 ± 0.01-fold of methylated *Cdh1*, 0.26 ± 0.07 fold of methylated *Atoh1* and 0.28 ± 0.07 fold of methylated *Pou4f3* expression in treated MUCs. ^*^ Indicates significant difference comparing with the control group.

To study whether 5-aza treatment was related to the generation of hair cell-like cells, we performed a genomic methylated DNA quantification assay to determine the genomic methylation level. The relative quantification of genomic methylated DNA (5-mC%) of control MUCs was 0.35 ± 0.04%. After 5 days of 5-aza treatment, the relative 5-mC% of treated MUCs decreased to 0.25 ± 0.04% (Figure 4A). Therefore, the genomic methylation inhibition showed 28.57% [(0.35 − 0.25%)/0.35 × 100%] decrease in genomic methylation level in response to 5-aza treatment. The statistical analysis indicated significant difference of relative 5-mC% between the control and treatment groups (*P* < 0.05, Student's *t*-test).

RT-PCR and quantitative PCR were performed to exam gene expression changes of DNMTs in the control and 5-aza-treated MUCs. After 5-aza treatment, expression of maintenance DNA methylatransferase *Dnmt1* and *de novo* DNA methyltransferase *Dnmt3a* was not significant changed in RT-PCR and quantitative PCR (Figures 4B,D). But expression of *de novo* DNA methyltransferase *Dnmt3b* was decreased in RT-PCR (Figure 4D), which was confirmed by quantitative PCR (0.52 ± 0.01 fold down-regulation; *P* < 0.05, Student's *t*-test; Figure 4B).

To study whether 5-aza affected the activity of DNMT, the DNMT activity assay was applied. The activity of DNMT in the control group was 70.33 ± 5.24 RFU/h/μg, but it reduced to 59.47 ± 3.87 RFU/h/μg after treatment (Figure 4C). The DNMT inhibition assay suggested that there was an approximately 15.37 ± 0.09% inhibition effect on DNMT activity after 5-aza treatment, which was statistically significant (*P* < 0.05, Student's *t*-test).

In the protein assay, both western blotting and immuno-fluorescence showed down-regulated expression of DNMT1 after treatment (Figures 4E,F). The immunofluorescence study indicated that DNMT1 expression was significantly decreased after treatment. Additionally, expression of DNMT1 was mainly showed in the cytoplasm of control MUCs, whereas its expression translocated to the nuclei of treated MUCs (Figure 4F). Overall, the DNMT1 expression change of treated MUCs showed in the protein level but not in the gene level.

### Mechanism of sensory hair cell-like cell differentiation following 5-aza treatment

It is known that 5-aza is able to induce genomic demethylation to regulate gene expression in some cell lines (Schneider-Stock et al., 2005; Song et al., 2014); however, it remains unclear whether 5-aza is able to affect the methylation level and plays a role in inducing expression of sensory hair cell genes in tissue specific stem/progenitor cell model MUCs. To address this question and also to further study the relationship between expression of sensory hair cell markers and 5-aza treatment, nested methylation specific PCR (nested-MSP) was performed to examine methylation pattern changes in the promoter region of sensory hair cell genes *Atoh1, Pou4f3*, and *Cdh1* (Figure 4G). The same amount of bisulfite converted gDNA of control and treated MUCs was used as templates in nested-MSP, and the primers were designed to specifically target to the CpG island in the promoter region of studied genes. According to the electrophoresis analysis of nested-MSP, the normalized amount of unmethylated *Cdh1* of the control and treatment group were similar, whereas the amount of methylated *Cdh1* of treated MUCs was slightly lower than that of control MUCs. In the study of nested-MSP of hair cell genes, *Atoh1* was highly methylated in control MUCs. After treatment, the amount of methylated *Atoh1* was significantly decreased, whereas the amount of unmethylated *Atoh1* was remarkably increased. Moreover, treated MUC exhibited an obviously higher expression of unmethylated *Atoh1* than methylated *Atoh1*. Another hair cell gene *Pou4f3* exhibited a significant high amount of methylated expression in control MUCs, whereas the amount of unmethylated *Pou4f3* was scarce. After treatment, expression of methylated *Pou4f3* was completely shut down, whereas the amount of unmethylated *Pou4f3* was significantly increased. The nested-MSP data suggested that 5-aza caused demethylation of *Atoh1* in the promoter region, which may contribute to increased expression of *Atoh1* gene.

To further examine the methylation level of studied genes in treated MUCs, methylated DNA immunoprecipitation (MeDIP) and quantitative PCR were performed (*n* = 3). Genomic DNA of the control and treated MUCs was randomly broken into DNA fragments using sonication. The same amount of DNA fragments was added to immunoprecipitation reaction, followed by quantitative PCR to analyze the quantity of methylated *Cdh1*, methylated *Atoh1* and methylated *Pou4f3*. The primers were designed to specifically target to the promoter region of studied genes. In control MUCs, the Cq value of methylated *Cdh1* was 27.35 ± 0.01, whereas it increased to 27.52 ± 0.46 after treatment, which was not statistically significant (*P* > 0.05, Student *t*-test). The Cq value of methylated *Atoh1* in control MUCs was 35.38 ± 0.38, which was increased to 37.33 ± 0.07 after treatment (*P* < 0.05, Student *t*-test). The Cq values of methylated *Pou4f3* were 23.00 ± 0.07 and 24.84 ± 0.17 in control and treatment group respectively (*P* < 0.05, Student's *t*-test). Compared to control MUCs (normalized to 1), MeDIP quantitative analysis using the 2^−ΔΔCq^ (Livak) Method showed 0.89 ± 0.01 fold of methylated *Cdh1*, 0.26 ± 0.07 fold of methylated *Atoh1* and 0.28 ± 0.07 fold of methylated *Pou4f3* in treated MUCs (Figure 4H). These studies suggested that decreased amount of methylated *Atoh1* and *Pou4f3* may lead to up-regulated hair cell gene expression, and that increased expression of hair cell gene *Atoh1* and *Pou4f3* was likely caused by demethylation of their promoter regions.

## Discussion

In this study, 5-aza was used to treat mouse utricle sensory epithelia-derived progenitor cell MUC. After the treatment, expression of prosensory genes, epithelial genes and hair cell genes was up-regulated. We found that 5-aza treatment caused increased protein expression of E-cadherin, Myosin VIIa, Myosin VI, and Pou4f3. FM1-43 permeation assay revealed that treated MUCs possessed the permeability of functional mechanotransduction channels. 5-aza significantly decreased DNMT1 protein expression and DNMT activity, by which the genome of MUCs underwent a remarkable demethylation process. Genomic methylation assays revealed that increased expression of sensory hair cell markers *Atoh1* and *Pou4f3* were likely resulted from DNA demethylation in their promoter regions.

5-aza is a DNMT inhibitor, which causes DNA demethylation by blocking DNMTs. Our study demonstrated that treated MUCs showed no obviously gene expression change of maintenance methyltransferase *Dnmt1* and *de novo* methyltransferase *Dnmt3a*, but down-regulated expression of *de novo* methyltransferase *Dnmt3b*. However, the protein expression of DNMT1 was significantly decreased, and DNMT activity was remarkably reduced following 5-aza treatment. Down-regulated DNMT1 protein expression and decreased DNMT activity may contribute to the demethylation of MUC genome. Our result is consistent with previous studies, which suggested that DNA demethylation was caused by down-regulated *Dnmt* gene and protein expression in cervical cancer, prostate cancer and acute leukemia cell lines (Chen et al., 2003; Walton et al., 2008; Lund et al., 2014). Additionally, our immunofluorescence study showed that DNMT1 mainly expressed in the cytoplasm of control MUCs, whereas its expression was reduced and translocated to the nuclei of treated MUCs. It has been reported that the intracellular localization of DNMT1 is various and is dependent on cell type, developmental stage, and pathological condition (Doherty et al., 2002; Ratnam et al., 2002; Lundberg et al., 2009; Desplats et al., 2011). In the current observation, DNMT1 translocation was observed in treated MUCs, but the mechanism of intracellular translocation is still obscure, which needs further investigation in our future studies. Overall, down-regulated DNMT1 protein expression and decreased DNMT activity may cause a remarkable demethylation in the genome of 5-aza-treated MUCs, which may contribute to the gene expression changes as discussed below.

Recent studies suggested that DNA demethylation may play an important role in stem cell reprogramming (Bagci and Fisher, 2013; Wongtrakoongate, 2015). For example, a differentiated cell line, normal adult basal prostatic epithelial (E-PZ) cells, could be reprogrammed and dedifferentiated into induced pluripotent stem cells by forced expression of embryonic pluripotent genes including *Sox2, Oct4*, and *SSEA-3* (Zhao et al., 2013). The authors studied the methylation level of pluripotent genes and prostatic epithelial genes and found that during prostate differentiation, the methylation level of pluripotent genes elevated, whereas the methylation level of prostatic epithelial genes reduced, suggesting that DNA methylation plays an important role in stem cell differentiation (Zhao et al., 2013). In the present study using a tissue specific progenitor cell line MUC, we found that DNA methylation level was related to expression of prosensory genes such as *Sox2* and *Lfng*. HMG-box transcription factor *Sox2* is essential for sensory progenitor development in the mammalian inner ear including the differentiation of hair cell precursors. *Sox2* positive prosensory cells acquired an active expression of *Atoh1*, a transcription factor critical for hair cell differentiation, therefore determining the cell fate as hair cells (Locher et al., 2013). Overexpression of *Sox2* in mouse otocyst induced sensory region specification, which subsequently stimulated prosensory cell differentiation and hair cell formation (Pan et al., 2013). It was reported that O-fucosylpeptide 3-beta-N-acetylglucosaminyltransferase, *Lfng*, was expressed in chicken otocysts and involved in the development of sensory organ in the inner ear development (Cole et al., 2000; Pujades et al., 2006). In the present study, 5-aza-induced DNA demethylation is likely to up-regulate expression of prosensory genes *Sox2* and *Lfng*, which may be involved in guiding MUCs along a development program toward hair cell formation. Additionally, we observed other prosensory genes, including *Bmp4* and *P27*^*kip*1^, changed their expression following treatment, suggesting that these prosensory genes may also be involve in the differentiation of MUCs.

In this study, control MUCs did not express epithelial genes *Cdh1, Krt8*, or hair cell gene *Myo6, Atoh1*, and *Pou4f3*. After 5-aza treatment, expression of these studied epithelial sensory hair cell genes was significantly increased. Protein expression of these epithelial sensory hair cell markers was elevated in treated MUCs in immunofluorescence and western blotting. FM1-43 permeation assays indicated that treated MUCs were permeable to FM1-43, which suggested that treated MUCs may possess some properties of mechanotransduction channels of sensory hair cells. Furthermore, the genomic methylation level of treated MUCs was significantly decreased. Expression of DNMT1 protein and the activity of DNMTs were down-regulated following 5-aza treatment. To understand whether DNA methylation level influences hair cell gene expression, MSP, and MeDIP using primers targeting the promoter regions of hair cell genes were performed. The results revealed that relative expression of methylated *Cdh1, Atoh1*, and *Pou4f3* was decreased and unmethylation of the promoter region of these genes was obviously increased after 5-aza treatment, which likely led to demethylation of the promoter region of studied genes, subsequently stimulated MUCs to upregulate expression of sensory hair cell genes and proteins, and finally contributed to differentiation of MUCs into hair cell-like cells. Our observation suggested that 5-aza treatment may be an approach to affect the methylation level of MUCs, and subsequently stimulates MUCs to differentiate into functional hair cell-like cells.

Compared to 5-aza-CdR that was used in our previous report (Zhou and Hu, 2015), 5-aza seems to have better effects on sensory hair cell induction. In comparison of relative gene expression, majority of hair cell genes showed significant changes after 5-aza treatment. For instance, *Atoh1, Espn*, and *Pou4f3* had 3.74 ± 1.27, 4.14 ± 0.07, 274.42 ± 45.49 fold increase after 5-aza treatment, but they did not show significant changes after 5-aza-CdR treatment. Further, 5-aza treatment stimulated hair cell protein expression including Myosin VIIa, Myosin VI, Pou4f3, and Math1, but 5-aza-CdR treatment did not cause obvious hair cell protein expression changes. Although 5-aza may cause adverse effects; however, no serious clinical complications and death attribute to 5-aza. Therefore, 5-aza has a preferable safety data and it has been approved by The Food and Drug Administration (FDA) for clinical therapy since 2004 (Kaminskas et al., 2005). Based on these data, 5-aza treatment seems to exert a better role in inducing hair cell generation from MUCs.

In summary, the DNMT inhibitor 5-aza caused DNA demethylation in the entire genome of MUCs to induce cell lineage specification. In the treatment group, gene and protein expression of sensory hair cell markers was remarkably increased. The FM1-43 permeability assay indicated that treated MUCs may possess some properties of mechanosensory transduction channels, suggesting that treated MUCs had differentiated into hair cell-like cells. Nested-MSP and MeDIP-quantitative PCR revealed that up-regulation of sensory hair cell genes was likely caused by demethylation of the promoter region of these genes. Our study demonstrated a novel epigenetic approach in stem cell biology, which could induce tissue specific inner ear progenitors to differentiate into functional sensory hair cell-like cells. In the meantime, it opens avenues to develop strategies to guide tissue specific stem/progenitor cells to become functional cells without altering DNA sequence, which is fundamental to the design of future clinical trials involving human cells.

## Author contributions

YZ and ZH designed the experiment, collected data, analyzed data, and wrote the main manuscript text. Both authors reviewed and final approved the manuscript.

## Funding

This study is supported by R01DC013275 from NIDCD/NIH.

### Conflict of interest statement

The authors declare that the research was conducted in the absence of any commercial or financial relationships that could be construed as a potential conflict of interest.

## Abbreviations

MUC: adult mouse utricle sensory epithelial derived progenitor cells
5-aza: 5-azacytidine
MeDIP: methylated DNA immunoprecipitation
MSP: Nested-methylation-specific PCR.
